# Frailty-Preventing Effect of an Intervention Program Using a Novel Complete Nutritional “COMB-FP Meal”: A Pilot Randomized Control Trial

**DOI:** 10.3390/nu15204317

**Published:** 2023-10-10

**Authors:** Takuo Nakazeko, Naohisa Shobako, Nobuhiko Shioya, Yoshitaka Iwama, Yukio Hirano, Shintaro Fujii, Futoshi Nakamura, Keiko Honda

**Affiliations:** 1 Future Food Research & Development Division, Nissin Foods Holdings Co., Ltd., Tokyo 192-0001, Japanyukio.hirano@nissin.com (Y.H.);; 2 KSO Corporation, Tokyo 105-002, Japan; 3 Nihonbashi Cardiology Clinic, Tokyo 103-001, Japan; 4 Laboratory of Medicine Nutrition, Kagawa Nutrition University, Saitama 350-0214, Japan

**Keywords:** randomized control trial, frailty, diet treatment, complete nutrition

## Abstract

Frailty is a huge concern for the aging population, and dietary nutrition is considered a key factor in the prevention of aging. To solve the problem of frailty in the aging population, we developed a novel dietary intervention program using a novel COMpletely Balanced for Frailty Prevention (COMB-FP) meal, based on the Dietary Reference Intake for Japanese; in addition, we conducted a pilot randomized control trial comparing an exercise program only (control group) with exercise plus the COMB-FP meal program (test group). We included 110 male and female healthy volunteers with pre-frailty or frailty; the trial lasted for 12 weeks. Two daily meals were replaced with the COMB-FP meals during the trial in the test group. Walking speed and cognitive function were significantly improved in the test group compared with the control group. We observed a similar pattern in other frailty-related outcomes, such as occupancy of the microbiome, World Health Organization well-being index (WHO-5), and oxidative stress. Our study might indicate the importance of a well-balanced intake of nutrients for frailty prevention.

## 1. Introduction

With the aging population worldwide, especially in developed countries, there is a corresponding increase in the prevalence of age-related conditions, and frailty is one of the important associated issues [1]. Frailty is a complex and diverse combination of diseases, including physical dysfunctions such as sarcopenia, poor bone health, and oral disfunction and psycho-social problems such as cognitive dysfunction, reduced mobility, and a lack of quality of life (QOL) [1]. Frailty can be considered a consequence of the normal aging process and can fluctuate between different states of severity [2]. According to a previous nationally representative survey, 8.7% of Japanese elderly people (≥65 years) were estimated as being frail and 40.8% were found to be pre-frail [3]. Similar trends were observed in surveys conducted on a global scale [4]. To solve the problem of frailty, some intervention programs, such as physical activity, on frailty were determined. In previous studies, physical activity and resistance training interventions improved physical performance and cognitive function [5,6,7]. As described in these studies, exercise interventions might have some effect on the prevention of frailty.

The association of dietary nutrition intake with frailty is also well studied, and malnutrition is considered one of the key factors in frailty [8]. Approximately 23% of older adults were reported as being malnourished or at risk of malnutrition [9,10]. Numerical factors, such as energy intake, should be considered when talking about malnutrition. The InCHIANTI study showed that a daily energy intake of ≤21 kcal/kg was associated with frailty; in addition, the amounts of protein intake, vitamin D, vitamin E, and folate were important [11]. The KNHANES-IV study, conducted in South Korea, may also support the importance of energy and micronutrients [12]. Simple dietary interventions do not yield a direct preventive effect on frailty. For example, in a randomized controlled trial (RCT) design [13,14,15] and its subsequent meta-analysis [16], protein and energy supplementation did not show strongly improving effects. Similarly, vitamin D and omega-3 fatty acid supplements did not provide protection against frailty [17]. Nutritional education encompassing whole-diet nutrients for pre-frailty participants was reported to prevent the progression of frailty [18]. These results emphasize the importance of studying whole-diet nutrients. Although numerous studies have reported on the relationship between intestinal bacteria and frailty, it is still too early to say with certainty whether there is a cause or effect [19]. However, some strains such as *Blautia*, *Fusobacterium*, *Lactobacillus*, and *Bifidobacterium* have been discussed for their roles in aging [20,21,22].

Thus, dietary intervention, and not intervention with supplements of only a few nutrients, might be a reasonable treatment for frailty. A popular dietary intervention for frailty is the “Nu-age study”, a large-scale RCT involving thousands of participants. Participants in the intervention group were well educated on the essentials of the Mediterranean diet (a guideline consisting of 14 kinds of foods, such as whole grains, vegetables, meat, nuts, oil, alcohol, sweets, and others) and received vitamin D supplements [23]. Cognitive function, one of the components of frailty, was improved by the intervention; however, its significance was only observed in the high-adherence group [24]. Another study found that the combination of exercise and a milk fat globule membrane improved frailty-associated outcomes [19]. In trials involving frail patients (i.e., those who were close to pre-frail, such as those with unintended weight loss), nutritional education, exercise, and their combination were examined [25]. This study found that nutrition education alone could not prevent frailty.

We previously reported on a novel dietary intervention program using the COMpletely Balanced meal (“COMB meal”), which included 33 kinds of nutrients, basically referring to guidelines in Japan [26]. This program involves the consumption of a diet regulated by nutrient content rather than ingredients, as in the Mediterranean diet. The dietary intervention of the COMB meal showed a hypotensive effect and improvement in glucose metabolism in an open-label RCT design [27]. In a preliminary trial of a single-arm intervention in the company’s cafeteria, protective effects on presenteeism and gut flora were observed [26]. These findings were also confirmed in an RCT (the manuscript is under review). In this study, we proposed a novel intervention program. It comprised a physical exercise lesson and meals adjusted to meet the recommended nutrient amount for older people (Table 1, Supplementary text). This study aimed to compare our novel program with an exclusive dietary intervention in determining the impact of meals related to frailty. Thus, we used a group undergoing an exercise program as a control group. We evaluated gut flora, QOLs, and biomarkers such as reflecting oxidative stress, associated with frailty in this pilot RCT.

## 2. Materials and Methods

The present study was designed and conducted according to the CONSORT 2010 statement guidelines, and a complete copy of the checklist is provided in Supplementary Table S1.

### 2.1. Study Design

The study was a 12-week, open-label, randomized controlled trial conducted under the principles of the Declaration of Helsinki, with the approval of the ethics review board of the Ethical Committee of Nihonbashi Cardiology Clinic (NJI-021-10-01), and according to the ethical guidelines for human research (Ministry of Education, Culture, Sports, Science and Technology; Ministry of Health, Labor and Welfare; Ministry of Economy, Trade and Industry Japan). The trials were registered in the UMIN Clinical Trials Registry (UMIN000046306).

### 2.2. Study Popuration

Residents from the suburbs were recruited to participate in this study through a registered monitor, administered by L-Smile Corporation from 11 to 29 November 2021. Interested participants were invited to the designated conference room (Tokyo, Japan). The details of the study and potential risks were explained, and written informed consent was obtained.

The inclusion criteria of this trial were as follows:

- Males and females aged 60 years and above.
- Individuals who were classified as having pre-frailty or frailty according to the revised Japanese version of the Cardiovascular Health Study (J-CHS) standards.
- Individuals with a cognitive function test result ranging from normal to mild cognitive impairment (MCI).
- Individuals who could take a test meal twice a day
- Individuals who had received a full COVID-19 vaccination (took second shots and/or booster shots).
- Individuals who could provide a sufficient explanation of the purpose and content of the research, have the ability to provide consent, demonstrate a proper understanding of the subject, voluntarily apply for participation, and agree to participate in writing.

The exclusion criteria were as follows:

- Individuals who planned to donate blood during the trial or donated within the past four weeks.
- Individuals at risk of developing allergies due to test meals.
- Individuals with implantable medical electrical equipment, such as pacemakers or other metal medical equipment, or the absence of limbs
- Individuals requiring care, the presence of motor dysfunction, dementia, and an inability to perform the exercise program.
- Individuals with a history of gastrointestinal surgery or a severe digestive disorder.
- Individuals judged to be extremely picky eaters and have dysphagia or a small appetite.
- Individuals with extremely irregular eating habits.
- Individuals with a history of regular intake of protein supplements.
- Individuals undergoing exercise therapy or diet therapy.
- Individuals with excessive alcohol consumption (60 g/day) or heavy smoking (21 cigarettes/day).
- Individuals with irregular daily routines owing to night work or working shifts.
- Individuals consuming of foods for specific health issues, functional foods, and supplements that would affect the trial.
- Individuals without a microwave oven.
- Individuals deemed ineligible for participation by the principal investigator based on blood test results.
- Participation in other research that involves the consumption of other test foods or the use of pharmaceuticals and cosmetics within one month of providing informed consent or showing willingness to participate.
- Individuals judged ineligible by the principal investigator.

### 2.3. Randomization

Participants were randomly assigned into two groups using the block randomization method, stratified by grip strength, walking speed, bone density, result of the cognitive function test, amount of daily energy and protein intake measured using the food frequency questionnaire (FFQg) [28], age, and sex. Randomization was performed by a chief of statistical analysis who was independent of the investigator.

### 2.4. Interventions

The trial was conducted in Tokyo, Japan, from 22 January 2022 to 24 April 2022. Participants assigned to the test group were instructed to replace two meals with the test meals and continue their usual diet at other times. The 33 types of nutrition for all test meals were adjusted within the range shown in Table 1. An example of the nutritional component of a test meal is shown in Table 2. All menus tested for this trial are listed in Supplementary Table S2. The menu items were consumed in any order. Participants in the control group were instructed to consume their usual diet. There were no restrictions on snacks or alcohol consumption. Participants were asked to record the following information in a diary every day: meal records (intake rate of the test meal was calculated to determine compliance); increase or decrease in food intake per day compared to the value prior to study participation; presence or absence of changes in physical condition and living conditions (including whether or not a home exercise program has been implemented, as described below); amount of alcohol consumed; number of medications; defecation statues; and any symptoms observed. In addition, participants were required to wear an activity monitor (EZ-064; Tanita, Tokyo, Japan) that could record the total burned and active calories, and the results were reported in the diary.

Participants assigned to both the control and test groups underwent the exercise program. All participants were required to join the group lesson of resistant exercise, as instructed by an exercise therapist, at weeks 1, 4, 8, and 10. In all exercise lessons, an exercise therapist was on site to supervise and guide the participants’ exercise. They were instructed to perform the same 20 min resistance exercise at home 3–5 times per week, as taught in the lecture and documented in the provided textbook. An exercise movie was also prepared, and the participants were instructed to watch it while exercising at home. Details of the exercise program are described in the Supplementary Materials.

### 2.5. Outcomes

The primary outcomes were grip strength, leg strength, walking speed, bone density, and cognitive function. The secondary outcomes were muscle mass, muscle rate, muscle mass by region (trunk, right or left arm, and leg), and frailty. In addition, we measured the following as exploratory outcomes: gut flora, WHO-5 (measuring feelings of happiness as described in the next section), and serum total antioxidant status (STAS). Since this is the first study to survey the effect of COMB-FP meals on this age group, we positioned this study as a pilot study with multiple outcomes.

### 2.6. Procedures

Grip strength was measured using a digital hand dynamometer, T.K.K. 5401 (Takei Scientific Instruments, Niigata, Japan). Leg strength was measured using T.K.K. 5715 and D.T.K.K. 5710e (Takei Scientific Instruments). Walking speed was measured via the modified method described previously [29]. Participants were asked to walk 14 m in a straight line and the time required to walk from the 2 m point to 1the 2 m point was measured. Bone density was measured by means of venues evo (Shibuya Corporation, Ishikawa, Japan). The Memory Performance Index (MPI) was used to assess cognitive function using the Japanese version of the MCI screen (Mirenia corporation, Tokyo, Japan). Muscle mass, muscle rate, muscle mass by region, body weight, and body fat rate were measured via bioelectrical impedance analysis using InBody 470 (InBody Japan, Tokyo, Japan). Frailty was measured using the Kihon Checklist [30], proposed by the Ministry of Health, Labour, and Welfare, and the revised J-CHS criteria [31]. Blood pressure was measured using the HEM-907 (Omron Healthcare, Kyoto, Japan). An FFQ test measuring energy and protein intake was conducted during hospital visits for screening and week 12 measurement. We used calculation software for calculating them (Excel Eiyoukun, Version 6, Kenpaku-sha, Tokyo, Japan).

Gut flora was measured using “mykinso pro”, as previously reported [26,32]. The detailed procedure is described below. Fecal samples were collected using Mykinso fecal collection kits containing guanidine thiocyanate solution (Cykinso, Inc., Tokyo, Japan) and were stored at 4 °C. DNA extraction from the fecal samples was performed using an automated DNA extraction machine (GENE PREP STAR PI-1200A, Kurabo Industries Ltd., Osaka, Japan) according to the manufacturer’s protocol. The V1–V2 region of the 16S rRNA gene was amplified using forward primer (16S_27Fmod: TCG GCA GCG TCA GAT GTG TAT AAG AGA CAG AGR GTT TGA TYM TGG CTC AG) and reverse primer (16S_338R: GTC TCG TGG GCT CGG AGA TGT GTA TAA GAG ACA GTG CTG CCT CCC GTA GGA GT) with KAPA HiFi Hot Start Ready Mix (Roche, Basel, Switzerland). To sequence 16S amplicons using the Illumina MiSeq platform, dual index adapters were attached using the Nextera XT Index kit. Each library was diluted to 5 ng/µL, and equal volumes of the libraries were mixed to 4 nM. The DNA concentration of the mixed libraries was quantified by qPCR with KAPA SYBR FAST qPCR Master mix (KK4601, KAPA Biosystems, Wilmington, MA, USA) using primer one (AAT GAT ACG GCG ACC ACC) and primer 2 (CAA GCA GAA GAC GGC ATA CGA). The library preparations were performed according to the 16S library preparation protocol of Illumina (Illumina, San Diego, CA, USA). Libraries were sequenced using the MiSeq Reagent Kit v2 (500 Cycles), with 250 bp paired ends. The paired-end reads of the partial 16S rRNA gene sequences were analyzed by using QIIME two (version 2020.8). The steps for data processing and assignment based on the QIIME 2 pipeline were as follows: (1) DADA2 for joining paired-end reads, filtering, and denoising; and (2) assigning taxonomic information to each ASV using the naïve Bayes classifier in the QIIME 2 classifier with the 16S gene of V1–V2 region data of SILVA (version 138) to determine the identity and composition of the bacterial genera.

Feelings of happiness were measured by means of the WHO-5 Well-Being Index [33]. Serum total antioxidant status was measured using an assay of 2,2-azino-bis (3-ethylbenzothiazoline-6-sulfonic acid) [34].

### 2.7. Statistical Analysis

As described in Section 2.5, we could not calculate a formal sample size due to the pilot nature of this study. We screened and randomized 110 patients, while the allocation of 55 or more patients per group in pilot RCT was recommended by Sim and Lewis [35,36]. Study parameters were presented as means ± standard deviation (SD). The efficiency analysis was based on per protocol set. The safety analysis was based on a modified intent-to-treat principle (full analysis set). 

To compare the numerical data for the control and test groups, the amount of change from 0 weeks was evaluated using an unpaired *t*-test (numerical data) or a Mann–Whitney U test. For all two-sided tests, the significance level was set at 5%. All analyses were performed using SPSS for Windows, version 27 (IBM Corporation, Armonk, NY, USA).

## 3. Results

### 3.1. Participants

**Figure 1:**
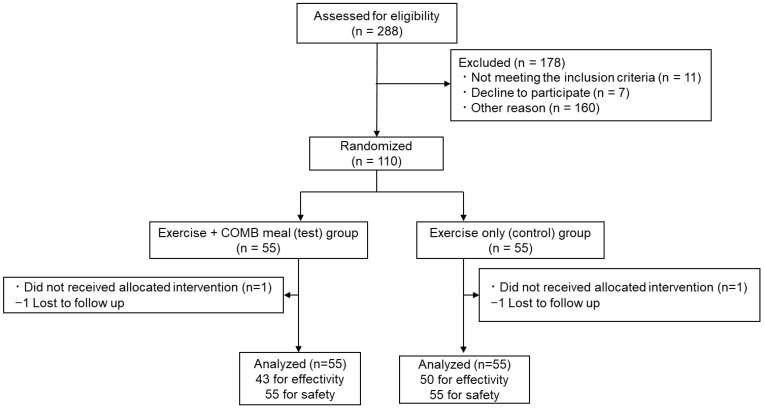
CONSORT 2010 flow diagram for study participants.

A total of 288 participants were recruited: 110 participants were enrolled and randomly assigned to the test (exercise and test food) or control (only exercise) group (Figure 1). Before further evaluation, two participants (one in the test group and one in the control group) withdrew for personal reasons unrelated to the trial. Data from participants whose nutritional surveys and changes in exercise habits and lifestyle were judged by the principal investigator, with the potential to interfere with the interpretation of the results, were excluded prior to statistical analysis of effectiveness. According to the diary kept by the participants, we confirmed that every participant consumed more than 90% of the test meals. The demographic characteristics of each group are shown in Table 3. The results of the FFQ indicated that changes in energy intake during the trial in both groups were relatively small, and there were no significant differences between the groups (Supplementary Table S3). Both groups also implemented the same degree of exercise programs (control group: 4.2 ± 0.8 times/week, test group: 4.1 ± 0.7 times/week; *p* = 0.555).

### 3.2. Effectiveness

The change in walking speed in the test group during the test period was significantly greater than that in the control group (Table 4). There was no significant difference in bone density (Table 4). The change in the MPI score in the test group was significantly higher than that in the control group (Table 4), thus indicating a significant improvement in cognitive function in the test group. There were no significant differences in other primary outcomes and secondary outcomes (Supplementary Table S4) between the two groups. 

The WHO-5 total score was significantly higher in the test group compared with that of the control group during the test period (Table 5). In the sub-questions, the answers to Q3 (“I have felt active and vigorous”) and Q4 (“I woke up feeling fresh and rested”) were significantly higher. 

Changes in the occupancy of the *Blautia* genus and *Anaerostipes* genus in the test group were significantly increased compared with those of the control group (Table 5). In contrast, the occupancy of *Fusobacterium* in the test group was significantly decreased compared with that of control group. There were no significant differences in the *Bifidobacterium* and *Faecalibacterium* genera between the two groups.

The change in STAS, the estimation of the global antioxidant rate [37], was significantly higher in the test group compared with that of the control group during the test period.

### 3.3. Safety

A total of 37 adverse events (12 in the control group and 25 in the test group) for 20 participants were reported. All reported adverse events are listed in Supplementary Table S5. The principal investigator determined that none of these adverse events were related to the test food.

## 4. Discussion

In the present study, we showed that our novel 12-week program, a combination of exercise and meal replacement with the “COMB-FP meal” (two meals per day), improved multiple outcomes related to frailty. Significant improvement in both functional ability and cognitive function was the unique effect of our intervention. Although our trial compared an exercise intervention to an exercise and dietary intervention, the importance of daily diet quality was emphasized.

Regarding the nutrients for frailty prevention, the importance of energy intake, protein, and micronutrients such as vitamins and omega-3 fatty acids was discussed [38]. Energy intake of 21 kcal/kg is considered the borderline for the risk of frailty [11]. According to the average participant weight, a goal of 1000 kcal (about 16.1 kcal/kg) was set, and the goal could be achieved by consuming approximately 300 kcal from the remaining meal. In terms of muscle weakness, protein supplementation is the most easily recalled intervention. Our intervention of two meal replacements per day guarantees a reliable daily protein intake of 46.4 g. The Dietary Reference Intakes for Japanese individuals recommend a maximum protein intake of 65 g/day for individuals over 50 years of age; our dietary intervention, which replaces two of the three meals, could fulfill at least 71% of the recommended value. The protein value provided by the intervention in the test group is considered adequate. However, considering the results of several trials, it is difficult to improve frailty in interventions lasting 12–24 weeks simply via protein supplementation [16]. The improvement in the test group might not be strongly related to protein supplementation, whereas 15–30 g of protein supplemented the normal diet in previous studies.

Some trials have verified the preventive effect associated with including only a few kinds of nutrients, such as micronutrients, on frailty prevention. Orkaby reported that intervention with vitamin D_3_ and omega-3 fatty acids could not reduce the risk of frailty. In contrast, Ng showed that supplementation with fiber, folate, vitamin B_6_, vitamin B_12_, and vitamin D could reduce the risk. One-year supplementation is required for significance [39]. Furthermore, this study showed that the combination of cognitive and exercise training and dietary interventions could shorten the duration to 12 weeks. Compared with nutrients, physical training might contribute more strongly to frailty prevention, according to Ng’s trial; it could significantly improve within 3 months and be sustained through to the end of the trial. Just for reference, we compared pre- and post-intervention values within each group, and grip strength was significantly increased in both groups (*p* < 0.01). In contrast to grip strength, walking speed was significantly improved by the dietary intervention (Table 4). Walking speed is one of the key components for judging frailty and is reported as a good predictor of frailty [40]; therefore, this result, shown in 12 weeks, might highlight the effectiveness of our novel dietary intervention program.

As reported by Jennings, the Mediterranean diet with vitamin D supplementation could not improve bone mineral density (BMD) [41]. In participants with osteoporosis at baseline, our study showed that the intervention attenuated the expected decline in femoral neck BMD. However, it had no effect on the lumbar spine or whole-body BMD. We did not measure BMD but rather the bone area ratio, which did not change significantly (Table 4).

As described in the Introduction, the Mediterranean diet might be one of the most studied whole dietary replacements for frailty, and the “Nu-age trial” might be the epoch-making study. As reported by Marseglia, one of the most popular results of this study is that cognitive function was not significantly improved by the Mediterranean diet with vitamin D supplementation [42]; however, its effectiveness was shown in the high-adherence subgroup. On the other hand, as described in Section 3.1, compliance with test meal consumption was relatively high, as every participant consumed more than 90% of the test meal. This high retention rate might be one of the advantages of our program.

As described in the Results, significant differences were not observed in the secondary outcomes. In addition, we plotted the relationship between the amount of energy or protein intake before the intervention and changes in the Kihon Checklist, representing the frailty after the intervention. However, as shown in Supplementary Figure S1, there was a correlation between energy (A) or protein (B) and frailty score. Moreover, when divided by the median, the lower group was significantly better than the higher group (*p* < 0.05). In other words, participants consuming a limited diet tended to improve their frailty using our dietary intervention. Previous studies on the importance of energy and protein intake might support our findings [43]. 

The “Nu-age trial” reported that continuous consumption of the Mediterranean diet changed microbiota and altered metabolism, including short-chain fatty acids [24]. In a previous animal study, the administration of fibers or some protein increased the abundance of *Blautia* species [35]. An observational study comparing frail and healthy older people showed that the occupancy of this genus was higher in healthy group [44]. Our results might support the positive benefits of this genus. The *Anaerostipes* genus was also significantly increased by our novel dietary intervention (Table 5). This genus is reported to ferment sugars, such as xylitol, and produce butyrate, considered beneficial for colonic health and the alleviation of colorectal cancer [45]. Positive effects have also been reported for the short-chain fatty acids produced by these intestinal bacteria; however, further research is needed. The relationship between occupancy of the *Fusobacterium* genus and frailty is not well known. A previous study of older people in Japan, comparing those who resided in nursing homes with those who were healthy, showed a higher *Fusobacterium* genus occupancy rate in the nursing home group [46]. Meanwhile, a positive correlation between colorectal cancer and occupancy has been reported [47]. The clinical benefits of the increased occupancy of *Fusobacterium* in our intervention warrant further studies in the future.

The WHO-5, known as the well-being index, was developed by the World Health Organization and comprises five questions [33]. In a previous study, the WHO-5 score was reported to have a relationship with depressive symptoms and anxiety [48]. Several studies examined the nutrients influencing the WHO-5 score. Yelverton reported that fiber, magnesium, niacin, thiamine, and folate were positively related to pregnancy [49]. Ugartmendia reported taking less soluble fiber as a factor of frailty for young male and female patients [50]. To our knowledge, this is the first report of WHO-5 and nutrients in older people; however, it is likely that WHO-5 score was improved by the supplementation of nutrients that were initially deficient. According to a large-scale cohort study, oxidative stress is related to frailty progression [51]. Our findings on STAS may warrant further in-depth study in the future.

Our study had six limitations. First, we could not clarify whether the functional substance contributed to the detected outcomes. We believe that an overall nutritionally balanced diet with adequate calories led to this improved outcome. However, further basic research is required to determine the key substances responsible for changes in outcomes. Second, we tested only Japanese participants. The Mediterranean diet has been tested on Westerners, and it remains to be verified whether this diet, as shown in Table 1 and Table 2, will have the same influence for Westerners. Third, we did not conduct in-depth research on the metabolites produced by the gut flora. It will be necessary to investigate the relationship between short-chain fatty acids, such as butyric acid, and outcomes in the future. Fourth, the current study was a non-rigorous intervention, consisting of two meals per day. Although this may have some value as an experimental pilot study, its effectiveness needs to be evaluated with more rigorous interventions in the future. Fifth, we determined outcomes with simple univariate analysis, and we did not adjust for any other factors. Finally, we should conduct another high-quality study with limited primary outcomes and sufficient power analysis for calculation of required participants. Since the expected impact in the real world is also uncertain, cohort studies might be needed in the future. However, we believe that this pilot RCT might reinforce the importance of nutrients based on the dietary reference of intake.

## 5. Conclusions

COMB-FP meals improved multiple outcomes related to frailty in elderly Japanese adults. Our results might indicate the important role of nutritional intervention in preventing frailty. In the future, it is important to study the extent to which the multiple outcome improvements from this intervention can contribute to the prevention of frailty. This study showed that the dietary intervention could improve several facets of frailty, such as cognitive function and QOLs.

## Figures and Tables

**Table 1:** Nutritional regulations of the COMB-FP meal used in the study.

Nutritional Regulations	/500 kcal		
		Lower Limit	Upper Limit
Protein	g	23.2	25.0
Fat	g	11.1	16.7
Saturated fatty acids	g		3.9
Carbohydrate	g	62.5	81.3
Fiber	g	7.5	
K	mg	893	
Ca	mg	268	424
Mg	mg	132	
P	mg	357	509
Ir	mg	2.7	8.5
Zn	mg	3.9	7.3
Cu	mg	0.32	1.19
Mn	mg	1.43	1.86
I	μg	46	509
Se	μg	11	76
Cr	μg	4	85
Mo	μg	11	102
RAE	μg	321	458
VD	μg	3.6	16.9
αTOC	mg	2.5	144.1
VK	μg	54	
VB1	mg	0.46	
VB2	mg	0.54	
Nia	mg	5.0	
VB6	mg	0.54	9.09
VB12	μg	0.9	
FA	μg	86	
PA	mg	2.14	
Biotin	μg	17.9	
VC	mg	36	
n6FA	g	3.6	
n3FA	g	0.9	
Salt equivalent	g		<3.0
Ile	mg	486	
Leu	mg	947	
Lys	mg	729	
Sulphur-containing amino acid	mg	364	
Aromatic amino acid	mg	607	
Thr	mg	364	
Trp	mg	97	
Val	mg	631	
His	mg	243	

K: potassium, Ca: calcium, Mg: magnesium, P: phosphorus, Ir: iron, Zn: zinc, Cu: copper, Mn: manganese, I: iodine, Se: selenium, Cr: chromium, Mo: molybdenum, RAE: retinol active equivalent, VD: vitamin D, αTOC: α-tocopherol, VK: vitamin K, VB1: vitamin B1, VB2: vitamin B2, Nia: niacin, VB6: vitamin B6, VB12: vitamin B12, FA: folic acid, PA: pantothenic acid, VC: vitamin C, n6FA: n6 fatty acid, n3FA: n3 fatty acid.

**Table 2:** Example of nutritional components of the test meal (grilled salmon with salt koji lunchbox).

Content	Unit	Amount	Content	Unit	Amount	Content	Unit	Amount
Energy	kcal	483	Se	μg	33	Biotin	μg	17.4
Protein	g	24.0	Cr	μg	3	VC	mg	36
Fat	g	15.7	Mo	μg	30	n6FA	g	3.5
Saturated fatty acids	g	3.0	RAE	μg	317	n3FA	g	1.6
Carbohydrate	g	61.3	VD	μg	10.1	Salt equivalent	g	2.2
Fiber	g	8.3	αTOC	mg	2.7	Ile	mg	1005
K	mg	863	VK	μg	52	Leu	mg	1797
Ca	mg	259	VB1	mg	0.46	Lys	mg	1767
Mg	mg	128	VB2	mg	0.53	Sulphur-containing amino acid	mg	991
P	mg	362	Nia	mg	12.3	Aromatic amino acid	mg	1839
Ir	mg	3.6	VB6	mg	0.52	Thr	mg	1028
Zn	mg	3.8	VB12	μg	3.5	Trp	mg	292
Cu	mg	0.31	FA	μg	83	Val	mg	1229
Mn	mg	1.38	PA	mg	2.07	His	mg	856
I	μg	45						

K: potassium, Ca: calcium, Mg: magnesium, P: phosphorus, Ir: iron, Zn: zinc, Cu: copper, Mn: manganese, I: iodine.

**Table 3:** Characteristics of participants.

	Unit	Control			Test			*p*
Male: Female		28	:	27	27	:	28	
Frailty: Pre-frailty		49	:	6	48	:	7	
Age	years	64.9	±	3.7	64.9	±	3.8	0.980
Grip strength	kg	28.7	±	9.0	27.7	±	8.3	0.532
Leg strength	kg	31.0	±	11.6	29.1	±	10.5	0.374
Walk speed	m/s	1.39	±	0.21	1.37	±	0.19	0.500
Bone density	%	26.6	±	3.4	26.9	±	3.4	0.687
MPI score	point	66.4	±	7.6	66.3	±	7.9	0.929
Energy intake	kcal/day	1984.0	±	559.1	2004.1	±	499.9	0.843
Protein intake	g/day	70.1	±	23.9	71.5	±	24.3	0.766
Body muscle mass	kg	41.0	±	7.7	41.1	±	8.2	0.979
Body muscle ratio	%	66.1	±	7.6	66.4	±	7.1	0.861
Trunk muscle mass	kg	19.3	±	3.8	19.2	±	4.1	0.871
Right arm muscle mass	kg	2.2	±	0.6	2.2	±	0.7	0.933
Left arm muscle mass	kg	2.2	±	0.6	2.1	±	0.6	0.736
Right leg muscle mass	kg	6.8	±	1.5	6.9	±	1.6	0.909
Left leg muscle mass	kg	6.8	±	1.5	6.8	±	1.5	0.998
Modified J-CHS score	point	1.9	±	0.7	1.8	±	0.7	0.582
Height	cm	162.2	±	7.9	163.0	±	8.5	0.593
Body weight	kg	62.3	±	10.7	62.0	±	11.5	0.910
BMI	kg/m^2^	23.6	±	3.4	23.2	±	3.0	0.489
Body fat rate	%	29.9	±	8.0	29.6	±	7.4	0.826
SBP	mmHg	133.3	±	15.7	135.2	±	13.7	0.491
DBP	mmHg	80.3	±	9.3	77.9	±	10.1	0.189
Pulse rate	beats/min	75.3	±	10.9	72.3	±	9.8	0.139
TG	mg/dL	92.4	±	48.1	104.5	±	70.2	0.294
HDL-C	mg/dL	67.9	±	15.7	72.2	±	20.5	0.223
LDL-C	mg/dL	130.6	±	26.1	133.6	±	30.5	0.581
Non-HDL-C	mg/dL	147.7	±	28.8	151.4	±	34.4	0.535
Fasting blood glucose	mg/dL	90.1	±	8.7	88.2	±	7.3	0.214
HbA1c	%	5.5	±	0.3	5.5	±	0.4	0.279

MPI: Memory Performance Index, J-CHS: Japanese version of Cardiovascular Health Study, BMI: body mass index, SBP: systolic blood pressure, DBP: diastolic blood pressure, TG: triglyceride, HDL-C: high-density lipoprotein cholesterol, LDL-C: low-density lipoprotein cholesterol, HbA1c: hemoglobin a1c.

**Table 4:** Evaluation of primary outcomes.

Physical function	unit		n	Week 0			Week 12			⊿	*p*
										95% CI	
Grip strength	kg	Control	50	29.3	±	9.5	30.3	±	8.9	0.3	0.607
		Test	43	28.1	±	9.0	29.4	±	9.1	(−0.8~1.3)	
Leg strength	kg	Control	46	34.7	±	11.7	33.5	±	13.3	1.4	0.387
		Test	39	33.5	±	11.5	33.7	±	12.1	(−1.8~4.6)	
Walk speed	m/s	Control	50	1.42	±	0.17	1.39	±	0.16	0.06	0.019
		Test	43	1.38	±	0.16	1.42	±	0.14	(0.01~0.12)	
Bone density	unit		n	Week 0			Week 12			⊿	*p*
										95% CI	
Bone density	%	Control	50	26.4	±	3.3	26.7	±	3.4	0.0	0.943
		Test	43	26.8	±	3.3	27.0	±	3.4	(−0.7~0.7)	
Cognitive function	unit		n	Week 0			Week 12			⊿	*p*
										95% CI	
MPI score	point	Control	49	65.8	±	7.2	66.4	±	7.3	2.4	0.038
		Test	43	65.0	±	7.4	68.1	±	6.9	-	

MPI: Memory Performance Index.

**Table 5:** Evaluation of other outcomes.

WHO-5	unit		n	Week 0			Week 12			⊿	*p*
										95% CI	
Total score	point	Control	50	15.4	±	4.3	16.1	±	3.5	1.6	0.027
		Test	43	14.3	±	4.3	16.6	±	3.4	-	
Q1	point	Control	50	3.0	±	1.0	3.3	±	0.9	0.2	0.264
		Test	43	3.0	±	1.0	3.4	±	0.8	-	
Q2	point	Control	50	3.3	±	0.8	3.5	±	0.8	0.3	0.073
		Test	43	3.2	±	0.9	3.6	±	0.8	-	
Q3	point	Control	50	3.1	±	1.1	3.2	±	0.9	0.5	0.013
		Test	43	2.7	±	1.0	3.3	±	0.9	-	
Q4	point	Control	50	3.2	±	1.1	3.2	±	1.1	0.4	0.040
		Test	43	2.8	±	1.1	3.2	±	0.9	-	
Q5	point	Control	50	2.8	±	1.2	3.0	±	0.8	0.2	0.337
		Test	43	2.7	±	1.2	3.1	±	0.9	-	
Occupancy of gut flora	unit		n	Week 0			Week 12			⊿	*p*
Genus										95% CI	
*Blautia*	%	Control	48	5.3	±	2.8	5.4	±	2.7	1.2	0.029
		Test	41	5.2	±	2.7	6.5	±	3.3	-	
*Anaerostipes*	%	Control	48	1.7	±	1.6	1.7	±	1.5	0.7	0.008
		Test	41	1.4	±	2.0	2.1	±	2.2	-	
*Fusobacterium*	%	Control	48	0.8	±	2.4	1.1	±	3.2	−0.6	0.037
		Test	41	1.1	±	2.7	0.7	±	1.9	-	
*Bifidobacterium*	%	Control	48	4.0	±	3.9	3.7	±	4.2	−0.3	0.556
		Test	41	3.8	±	3.8	3.2	±	3.6	-	
*Faecalibacterium*	%	Control	48	4.6	±	3.3	5.2	±	3.1	−0.4	0.397
		Test	41	5.3	±	3.3	5.5	±	3.5	-	
Antioxidant activity	unit		n	Week 0			Week 12			⊿	*p*
										95% CI	
STAS	μM	Control	50	1380.6	±	160.8	1371.5	±	148.1	53.2	0.003
		Test	43	1325.3	±	151.9	1369.3	±	148.2	(18.2~88.1)	

STAS: serum total antioxidant status.

## Data Availability

The researchers presented plans with appropriate methodologies and obtained consent from all co-authors.

## Supplementary Materials

The following supporting information can be downloaded at: https://www.mdpi.com/article/10.3390/nu15204317/s1, Supplementary text: information about exercise program. Supplementary Table S1. CONSORT2010 checklist. Supplementary Table S2: All menu items of the test meal. Supplementary Table S3: Changes of physical activity and energy intake during the test period. (A) Result of changes in activity monitor. (B) Result of changes in FFQ. Supplementary Table S4: Results of secondary outcomes. Supplementary Table S5: Reported adverse events. Supplementary Figure S1: Relationship between energy (A) or protein (B) intake and score of the Kihon Checklist, representing frailty in the test group. r: correlation coefficient.

## Abbreviations

αTOC: α-tocopherol, BMD: bone mineral density, BMI: body mass index, Ca: calcium, COMB-FP meal: COMpletely Balanced for Frailty Prevention meal, Cu: copper, Cr: chromium, DBP: diastolic blood pressure, FA: folic acid, FFQ: Food Frequency Questionnaire, HbA1c: hemoglobin A1c, HDL-C: high-density lipoprotein cholesterol, I: iodine, Ir: iron, J-CHS: Japanese version of Cardiovascular Health Study, K: potassium, LDL-C: low-density lipoprotein cholesterol, MCI: mild cognitive impairment, Mg: magnesium, Mn: manganese, Mo: molybdenum, MPI: Memory Performance Index, n6FA: n6 fatty acid, n3FA: n3 fatty acid, Nia: niacin, P: phosphorus, PA: pantothenic acid, RAE: retinol active equivalent, SBP, systolic blood pressure, Se: selenium, TG: triglyceride, STAS: serum total antioxidant status, VB1: vitamin B1, VB2: vitamin B2, VB6: vitamin B6, VB12: vitamin B12, VC: vitamin C, VD: vitamin D, VK: vitamin K, Zn: zinc.
