# Supplementary material for: Frailty-Preventing Effect of an Intervention Program Using a Novel Complete Nutritional “COMB-FP Meal”: A Pilot Randomized Control Trial
Source: Nutrients. 2023 Oct 10;15(20):4317. doi: 10.3390/nu15204317 (PMC10609752; doi:10.3390/nu15204317)
Supplement: Supplementary file 1 [file nutrients-15-04317-s001.zip › Supplemental_text.pdf]

Supplemental text1

Text of the exercise program distributed to the participants.

1. Knee circles (Gluteal muscles)
   ① Lie on your side and bend your knees to perpendicularly.
   ② Pull your leg and rotate your knees in a circular motion.

2. Inner thigh (Adductor muscle)
   ① Lie on your side and bend your knees to perpendicularly.
   ② Extend your knees and raise them straight up.

3. Raise your knees at right angles (gluteal region)
   ① Lie on your side and bend your knees to perpendicularly.
   ② Raise your knees straight up, keeping your knees and hip at right angles.

4. Elbow at 90 degrees (shoulder inner muscle)
   ① Hold the tube a little shorter than shoulder-width apart with the flat of the hand facing up.
   ② Slowly, in a parallel manner, spread the tube without recoil.

5. Shoulder stretch (back shoulder)
   ① Grip the tube shoulder-width apart with the flat of the hand down.
   ② Slowly, in a parallel manner, spread the tube without recoil.

6. Elbow bending (biceps brachii muscle, biceps)
   ① Grip the tube with the flat of the hand facing up.
   ② Bend your elbow about 90 to 100 degrees.

7. Squat (Whole legs)
   ① Spread your feet wider than hip-width apart and bend forward. The line of sight is on the floor.
   ② Stand up until your posture is upright.

8. Raising and lowering the heel
   ① Stand with your feet hip-width apart.

② Raise your heels with your back straight.
