# Supplementary figures and images for: Frailty-Preventing Effect of an Intervention Program Using a Novel Complete Nutritional “COMB-FP Meal”: A Pilot Randomized Control Trial
Source: Nutrients. 2023 Oct 10;15(20):4317. doi: 10.3390/nu15204317 (PMC10609752; doi:10.3390/nu15204317)

## Slide 1
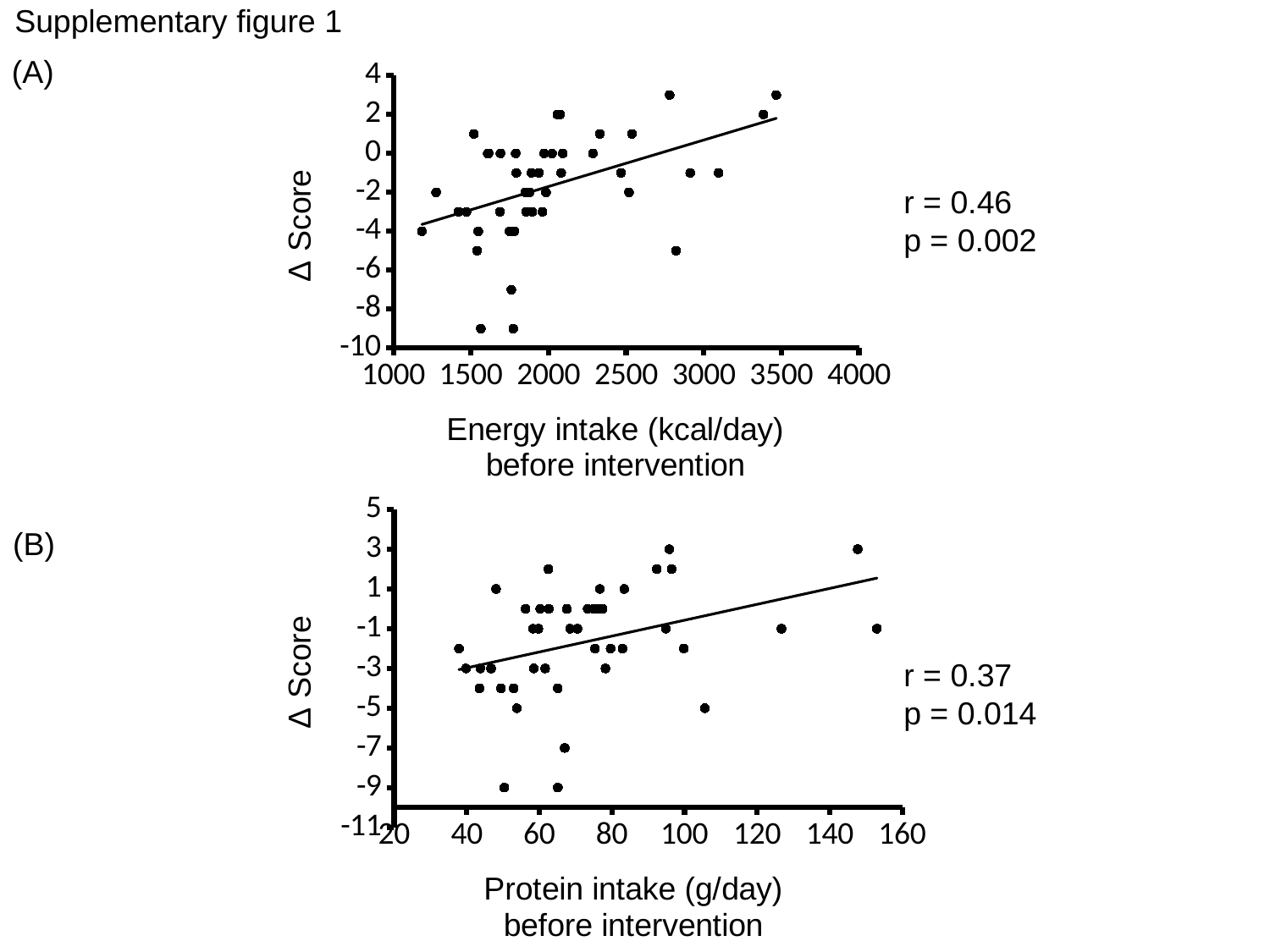

Supplementary figure 1
(A)
### Chart
| Category | 一般的分類 |
|---|---|r = 0.46
p = 0.002
### Chart
| Category | 一般的分類 |
|---|---|(B)
r = 0.37
p = 0.014

Supplement: Supplementary file 1 [file nutrients-15-04317-s001.zip › Supplementary_figure1.pptx]
